# Living arrangements, health lifestyles, and health outcomes among Chinese oldest-old

**DOI:** 10.3389/fpubh.2023.1235768

**Published:** 2023-10-09

**Authors:** Jie Wang, Lanxi Zhang, Shen Wang, Li Zhang

**Affiliations:** ^1^School of Law, Anhui Normal University, Wuhu, Anhui, China; ^2^Department of Sociology and Anthropology, School of Oriental and African Studies, University of London, London, United Kingdom; ^3^School of International Relations, Beijing International Studies University, Beijing, China; ^4^School of Sociology, China University of Political Science and Law, Beijing, China

**Keywords:** health lifestyles, living arrangement, latent class analysis, oldest-old, CLHLS

## Abstract

**Background:**

Prior literature has documented a strong correlation between living arrangements and older adults' health outcomes. However, few studies have explained why this association exists. This study took the health lifestyle theory approach and brought health lifestyles into the link between living arrangements and Chinese oldest-old health outcomes. It examined (1) whether healthy lifestyle behaviors among the oldest-old varied by household contexts and (2) whether the health disparities among the Chinese oldest-old in different household contexts could be partially explained by their healthy lifestyles.

**Methods:**

Using the most recent 2018 data released by the Chinese Longitudinal Healthy Longevity Survey (CLHLS), latent class analysis was applied to identify predominant health lifestyles among the Chinese oldest-old aged 85-105 years. Regression analyses were used to test the mediating effect of health lifestyles.

**Results:**

Three distinct classes representing the health lifestyles of Chinese oldest-old emerged; health lifestyle patterns were found to vary by elders' living arrangements. The respondent's health lifestyles in diverse residential structures served as a mediator which can partially explain the health disparities among the oldest-old.

**Conclusion:**

The results suggested that health lifestyles can serve as a mediator to explain the association between oldest-old living arrangement patterns and their health outcomes. The findings highlighted the importance of family, lifestyles, and cultural contexts to the health of the oldest-old.

## 1. Introduction

The linkage between living arrangements and older adults' health and wellbeing has been well-studied in social sciences. Different health outcomes have been considered, including subjective wellbeing, functional health, self-rated health (SRH), and mortality (1, 2). Researchers documented mixed results when studying health differentials of older adults who lived alone and who lived with others. Some found that co-residence created tension which was detrimental to older adults' health. Researchers showed living alone benefited older adults' mental health (3). While others challenged such results by showing co-residence was beneficial to older adults' health (4). Those who lived in institutions were found to have a higher mortality rate (5). Living alone has also been documented to lead to poor health conditions, including depression, cardiovascular disease, and dementia (6, 7). Researchers further emphasized that the beneficial effects of co-residing on older adults' health varied in different family contexts. Those who lived only with a spouse tended to report better health than those who lived with a spouse and children (8). Living with a married son was found to be more beneficial to parents' psychological wellbeing than living with other children in Vietnam; living with a daughter was found to bring greater benefits than living only with a son (9).

Despite many theoretical explanations accounting for the linkage between older adults' living arrangements and their health outcomes, few of them have considered the health lifestyle approach. Recent studies have proved health lifestyles to be an important factor that determines one's physical and overall health outcomes (10–12). Under this proceeding, this article intended to fill the voids of prior literature by bringing health lifestyles into the research scope to detect whether health lifestyles can serve as a mediator linking living arrangements and health outcomes among the Chinese oldest-old. In other words, co-residence or co-residing in various household contexts can lead to different health-enhancing lifestyles, such as an increased involvement in exercise, or a decreased consumption of cigarettes and alcohol, which in turn promotes older adults' health. This analysis focused on the oldest-old, those 85 years and above. This is because there has been growing recognition that the older adult population aged 65 years and older is heterogeneous. The study thus restricted its focus to the oldest-old, a fast-growing population in Chinese society. Relying on the latent analysis strategy, the study used the 2018 wave of the Chinese Longitudinal Health and Longevity Survey (CLHLS), a nationally representative data, to perform the analysis. Findings based on analyzing nationally representative data in China are also valuable to addressing health promotion-related issues among the oldest-old population in other Asian countries. Understanding how health behaviors cluster together among older adults in various household contexts can also help to expand theories of living arrangements and health disparities in general.

## 2. Literature review

### 2.1. Living arrangement and its association with Chinese older adults' health

China is a country that has a strong tradition of filial piety and an expectation of sons providing care to older adults; living alone is, therefore, less desirable as compared to Western countries. With drastic social and economic changes, expectations and preferences regarding living arrangements have changed in recent decades, especially in urban areas. The one-child policy and increased female labor force participation have also limited the younger generation's ability to provide care. Chinese society has provided alternate sources of old-age support as well. Some parents would prefer not to live with a married son if the situation allowed. Daughters are just as likely to provide support as sons (13). Thus, there has been a rapid increase in the number of senior households living alone or only with their spouses (14). Even with dramatic changes in Chinese society, traditional family norms have remained. An older adult co-residing with one or more adult children has still been a long-standing and continuing practice and a fundamental way of household structure in China (15). Living with sons has still been prevalent, even if living with daughters has become more desirable and more common (16).

Regarding the association between living arrangements and older adults' health outcomes, although mixed results have been documented in a variety of social contexts, in China, where there is a strong tradition of filial piety and an expectation of sons providing care to older adults, empirical findings largely corroborated that co-residence had a positive impact on older adults' health. In general, older adults who lived with adult children were found to report better SRH, a lower likelihood of having activity of daily living (ADL) disabilities, and were less likely to feel lonely or depressed (4, 17–19). Scholars also observed that Chinese older adults who lived with family members had greater odds of reporting good quality of sleep and a longer duration of sleep (20). By differentiating the Chinese oldest-old from those who lived with sons and those who lived with daughters, Chen and Short (13) further pointed out that the oldest-old who lived with daughters reported the best emotional health results, and those who lived alone displayed the worst health outcomes. Prior studies also showed that older adults who moved into an institution from living with family faced a greater risk of dying compared to those who continued to live with family members (5).

### 2.2. Mechanisms linking living arrangements and health

Several mechanisms have been proposed to explain the association between living arrangements and older adults' health outcomes. These theories can be categorized as follows:

#### 2.2.1. Social integration explanation

Scholars contended that co-residence promotes older adults' health because the household provides an important context to individuals for social integration and various dimensions of support. Co-residence with adult children meets the needs of daily care and provides physical and emotional support to older adults. Social support may buffer the harmful physical and mental health effects of exposure to stress and negative social events (21–23). Thus, co-residence promotes elders' health and wellbeing (24, 25).

#### 2.2.2. Filial piety theory

Researchers argued that in societies where there are patrilineal kinship systems, individuals are considered to belong to their father's lineage. Older adults are expected to co-reside with a married son and his wife. Patrilocality is considered a manifestation of filial piety or the moral obligation of children to revere their parents (26). In China, filial piety is a fundamental cultural expectation; going against it by living alone may cause feelings of stress, even guilt, for an elder person. Given that filial piety is the most important cultural ideal in Confucian Asian societies, living with married sons is likely to benefit older adults' health, especially psychological wellbeing (27, 28). Such a beneficial effect is considered independent of intergenerational support (9).

#### 2.2.3. The balance of resources and demands theory

Researchers holding this explanation argued that demands without corresponding resources may lead to poorer health. When resources equal or exceed demands, household relations may benefit or protect health (29, 30). According to this theory, a person's resources diminish as he or she grows older, which is characterized by a lower physical capacity to do IADL activities; while their demands in terms of requiring assistance and care gradually increase. Evidently, living alone can lead to a resource deficit because there is no social support in the household. The resources available through living with adult children vary according to how much time adult children are available at home and how much care adult children can provide to elder parents. In this sense, households with different living arrangement structures make different demands on individuals and offer different resources too. Thus, older adults who stay in household contexts can balance their resources and demands and are likely to have better health outcomes.

#### 2.2.4. Social conflicts theory

Those who supported living alone is beneficial to individual health contended that relations among household members can create tensions, conflicts, and negative interactions, which damage older adults' health and wellbeing (31). Thus, social conflicts between members of the household are considered an explanation of the health disparities among seniors living with others (5).

#### 2.2.5. Economic wellbeing as a mediator explanation

A substantial amount of literature suggests that living with family members results in an increase in economic wellbeing which promotes one's health status (32). This is because economic wellbeing improves the family's conditions; the sharing of financial and social resources makes the costs more economical. The gathering of wealth also protects against the risk of unexpected out-of-pocket medical and other spending. If living with family members is due to financial constraints but not by self-choice, then the opposite situation could occur.

### 2.3. The health lifestyle approach

Different from the above theories, the health lifestyle approach can be considered as a theoretical development in research of health disparities (10, 12, 33, 34). The benefit of this perspective is that it has extended the scope of existing analyses on health by merely looking at single health behaviors, such as poor dietary habits, sedentary lifestyle, cigarette smoking, and excessive alcohol consumption that have been commonly used in prior studies (35). Scholars argued that health behaviors tend to cluster in ways that reflect the social and structural contexts of individuals, which in turn affects individual health status (36). This is because behaviors are not isolative but co-occur with one another (34). Health lifestyle theories therefore contended that concentrating on single behaviors or small subsets of risky behaviors provides limited insight into health behavior patterns.

Previous studies have documented an association between living arrangements and older adults' health lifestyles. Chinese older adults who lived with family members were found to have greater odds of reporting good quality of sleep and a longer duration of sleep (20). Older adults who lived alone and who lived in a large household had a higher risk of inadequate fruit and vegetable intake (37). Older adults who lived alone were found to be more likely to have food insecurity problems and higher possibilities of smoking compared to those living with spouses/partners. It was pointed out that food insecurity, cigarette smoking, and alcohol drinking partially explained the differences in SRH due to living arrangements (38). Meanwhile, scholars also showed a strong link between health lifestyles and individual health outcomes, including mental health, cognitive function, SRH, longevity, and alike (39, 40).

Considering the association between living arrangements and health lifestyles, as well as the link between health lifestyles and health outcomes, that have been documented in the existing literature, this study hypothesized that health lifestyles may serve as a factor mediating the relationship between living arrangements and Chinese oldest-old health outcomes. The selection of health lifestyle as well as health status measures in this study was based on the commonly used measures in previous studies. The analysis answered two main questions: First, how do predominant health lifestyles of the Chinese oldest-old vary among different living arrangement settings? Second, do these main health lifestyles explain part of the health disparities due to the living arrangements of the Chinese oldest-old? The results based on analyzing the Chinese data were supposed to enrich theories explaining the health disparities due to older adults living arrangements in general. Below, the article moved to an introduction of data, measures, and methods used in the study.

## 3. Data, measures, and methods

### 3.1. Data

Data came from the 2018 Chinese Longitudinal Healthy Longevity Survey (CLHLS) which was conducted in randomly selected half of the counties/cities in 22 provinces of China. Until now, eight waves (1998, 2000, 2002, 2005, 2008, 2011–2012, 2014, and 2018) of survey data have been collected. The survey was initially launched to meet the needs for scientific research on the oldest-old. Thus, the dataset provided an excellent source for studying seniors in China. It was pointed out that persons who reported an age of 106 years or higher were considered invalid cases (41). Thus, persons aged 106 years and higher were not included in this study due to insufficient information to validate their reported extremely high age. The study eventually obtained 7,943 oldest-old aged 85 to 105 years, with 3,056 male and 4,887 female populations.

### 3.2. Measures

#### 3.2.1. Living arrangements

The classification of living arrangements fell in line with the classification used in prior literature (13, 42) and it was classified into four mutually exclusive groups. Under each group, the respondent was further classified into sub-groups (please see **Table 3** for details). The four mutually exclusive groups were as follows: (1) *Living with a spouse only*: older adults who were living only with their spouse; (2) *Living with adult children/grandchildren only*: Under these groups, respondents were classified into three sub-groups, which were (a) living with son(s) or grandson(s) only, (b) living with daughter(s) or granddaughter(s) only, and (c) living with both son(s) and daughter(s). Living with daughter-in-law and granddaughter-in-law was considered as living with son and grandson-in-law, respectively; living with son-in-law and grandson-in-law was considered as living with daughter and granddaughter, respectively; (3) *Living with a spouse and child(ren)/grandchild(ren)*: Those who lived with a spouse and child(ren) or grandchild(ren) only. Similarly, three sub-groups were classified under this group, including (a) living with a spouse and son(s) or grandson(s) only, (b) living with a spouse and daughter(s) or granddaughter(s) only, and (c) living with a spouse and both son(s) and daughter(s). Again, living with daughter-in-law and granddaughter-in-law was considered as living with son and grandson-in-law, respectively; living with son-in-law and grandson-in-law was considered as living with daughter and granddaughter, respectively; (4) *Living with no spouse, and no children*. Under this group, respondents were classified into three sub-groups, including (a) living alone, (b) living in nursing homes, and (c) living with others (e.g., nephew/niece, siblings, servants, other relatives, etc.).

The preliminary analyses showed that living with son(s)/grandson(s) did not have significant differences with living with daughter(s)/granddaughter(s) when predicting health lifestyle latent class memberships or the respondent's health status. Thus, when running regression analyses, we did not differentiate the respondents living with son(s)/grandson(s) from those living with daughter(s)/granddaughter(s). The living arrangement patterns were classified as six categories in regression analyses, i.e., in addition to the first three categories, the other three sub-categories under the fourth category were considered as the 4th, 5th, and 6th categories (**Table 3**).

#### 3.2.2. Health lifestyle measures

Health lifestyle measures used in previous analyses generally fell into the following categories:(1) dietary patterns (including eating fruits, vegetables, and breakfast), (2) smoking and alcohol consumption, (3) sleep, (4) obesity and physical activity, (5) seat belt wearing and media use, (6) body mass index (BMI), and (7) regular physical examination (10, 39, 43–48). Due to data constraints, the selection of health lifestyle indicators in this study was based on prior studies and mainly applied four key domains, including dietary behaviors, smoking and alcohol use, sleep, and physical and leisure activities.

The first domain was dietary behaviors. In the CLHLS survey, the respondent was asked about the frequency of eating fresh fruits, fresh vegetables, and drinking tea. Prior research pointed out that tea drinking is related to longevity and reduced risk of mortality and death from cardiovascular diseases (49, 50). Tea consumption was therefore considered to be an important health lifestyle behavior in this study. These three variables were coded as dichotomous ones labeling respondents answering “almost every day” as “1” and “0” if otherwise.

The second domain is related to smoking and alcohol use. Since the variables measuring the respondent's exact amount of cigarette or alcohol consumption had about 80.0% of responses as missing values, the research therefore applied other alternative measures. The study relied on CLHLS survey questions asking the respondent whether he or she smoked or drank alcohol “in the past” and “at present”. The respondent who smoked in the past and at present was coded as “1” and “0” if otherwise. The same rationale and coding strategy were also applied to the alcohol consumption variable.

Sleep was the third domain of health lifestyles given that sleep has been repeatedly treated as an important measure of health lifestyles in previous analyses (11, 34, 39). Sleep was measured by two variables: sleep duration and sleep quality. The sleep duration variable was coded as “1” if the respondent answered having 8 h or more sleep each day and “0” if otherwise. The sleep quality variable was dichotomized with those who reported their sleep quality as “good” and “very good” as “1”. Those who reported sleep quality as “so so”, “bad”, and “very bad” were considered as poor sleep quality and coded as “0”.

The fourth domain was physical and leisure activities. The research judged if the respondent was physically active by relying on two survey questions asking whether the respondent exercised regularly in the past and at present. Those who exercised regularly both at present and during the past were coded as “1” and “0” if otherwise. Leisure activities were classified into sedentary and active activities. Epidemiologic studies have utilized measures of moderate-vigorous intensity exercise to define active activity and highly prevalent sedentary behaviors such as television viewing (51). The measures of sedentary and active behaviors were chosen based on such a definition. Sedentary activities were such as reading newspapers/books, playing cards and/or mah-jong, and watching TV and/or listening to the radio. Raising domestic animals and doing gardening work were considered active activities. For those who participated in leisure activities almost every day were coded as “1” and “0” if otherwise.

#### 3.2.3. Health outcome measures

The selection of health outcome measures was based on how health status was operationalized in prior analyses. Although previous studies have applied a striking array of health outcome measures, these measures can largely be classified into four dimensions: (a) mortality, morbidity, and frailty, including chronic illnesses (52–56); (b) perceived health or self-rated health (10, 33, 38, 57); (c) functional health which is indicated as ADL and recurrent falling (58–62); and (d) mental health, such as physiological wellbeing, depression, and cognitive function (63–66). Although the CLHLS questionnaire did not include all of the above health outcome indicators, it did have questions asking about older adults' self-rated health, cognitive function, and subjective wellbeing. These measures are consistent with the above four dimensions of commonly used health outcome measures. In addition, these measures cover the main domains of health measures; therefore, they were used in his study to capture the health status of the respondents.

The respondent's *self-rated health* was coded as an ordinal variable (1 = very bad, 5 = very good). *The cognitive function* of the respondent was measured by using the Chinese version of the Mini-Mental State Examination (MMSE). The MMSE was adapted from Folstein, Folstein, and McHugh (67) and tested four aspects of cognitive functioning: orientation, calculation, recall, and language. The total possible score on the MMSE is 30, with lower scores indicating poor cognitive ability. Responses of “unable to answer” were coded as incorrect answers.

Regarding subjective wellbeing, the study relied on a number of CLHLS questions that measured the positive and negative feelings of the respondents, respectively. The questions asked about positive feelings included the following: (1) How do you rate your life at present? (2) Do you always look on the bright side of things? (3) Are you feeling energetic? (4) Are you full of hope for future life? (5) Are you happy now as when you were younger?

The questions asked about negative feelings included the following: (1) Are you ashamed, regretted, or guilty about what you have done? (2) Are you angry because you cannot get used to people or things around you? (3) Do you often feel that people around you are not trustworthy? (4) Are you worried about some small things? (5) Is it difficult to concentrate when you are doing things now? (6) Do you feel sad or depressed? (7) Do you feel the older you get the more useless you are? (8) Are you nervous or scared? (9) Do you feel lonely? (10) Do you feel unable to continue your life? (11) Do you feel uneasy, worried, and annoyed? (12) Do you feel that you cannot stop or cannot control worry? (13) Are you worried too much about all kinds of things? (14) Are you very nervous and it is difficult for you to relax? (15) Are you very anxious, so you cannot sit still? (16) Are you easy to get annoyed or easily irritated? (17) Do you feel like something terrible happened?

The responses ranged from 1 to 5 for the questions asking positive feelings with “1” representing always or very good and “5” representing never or very bad. For the first 10 questions measuring negative feelings, the responses ranged from 1 to 5 with “1” indicating the weakest feel and “5” the strongest feel. The coding scale for the rest of the questions measuring negative feelings was somewhat different with responses ranging from “1” to “4”. Similarly, “1” represented never and “4” represented almost every day. Thus the maximum positive and negative feeling scores are 25 and 78, respectively. Since CLHLS data were not collected to examine the psychological wellbeing of older adults, the above question may not be a perfect indicator of one's subjective wellbeing. However, Chen and Shot (13, p. 1388) indicated that “they represent important dimensions of subjective wellbeing, such as life satisfaction, happiness, and loneness.” Thus, measures associated with the above questions were considered as legitimate indicators of the oldest-old psychological wellbeing.

The response codes were summed, creating a range of 5 to 25 for the positive feeling score and 17–78 for the negative. The internal consistency coefficients for the two summed scores were alpha = 0.70 and 0.87, respectively. These values indicated that the two summed scores were valid and acceptable. The logic behind the strategy was that each group of variables measured the same concept. This strategy reduced the number of variables in the analysis and improved the efficiency of the regression models. After summing each set of variables to a single variable, Cronbach's alpha was applied to assess the reliability of a given set of variables (68).

#### 3.2.4. Control variables

The study also controlled for the respondent's demographic and socioeconomic characteristics, including age, gender, rural/urban residence, education, and per capita household income. The early childhood (or parental) socioeconomic status (SES) was also controlled because socioeconomic condition in early childhood was found to have a cumulative effect on one's later life health status and mortality (69, 70). Such measures were whether the respondent frequently went to bed hungry as a child and the education of the respondent's father. Table 1 shows descriptive statistics for all variables used in the analysis.

**Table 1:** Summary statistics for all variables: Chinese oldest-old aged 85–105 (*N* = 7,943).

**Variables**	**Mean (or %)**	**SD**	***N***
**Health lifestyle variables**			
1) R eats fresh fruit almost everyday	21.1		7,884
2) R eats fresh vegetables almost everyday	41.6		7,891
3) R drinks tea almost everyday	12.3		7,713
4) R smoked before and still smokes	10.2		7,943
5) R drank before and still drinks	9.7		7,943
6) R had good quality of sleep	52.9		6,808
7) If R normally sleeps at least 8 hours	53.5		7,290
8) R exercised regularly during the past and still regularly exercises at present	15.9		7,943
9) R participates active leisure activities frequently	27.1		7,943
10) R participates sedentary leisure activities frequently	40.4		7,943
**Health status variables**			
1) R's self-rated health (mean)	3.4	0.9	6,737
2) R's cognitive function score (mean)	26.8	3.8	2,079
3) R's positive feeling score	12.7	3.4	5,510
4) R's negative feeling score	29.0	7.5	5,050
**Control variables**			
**R's characteristics**			
Age (mean)	94.7	5.9	7,943
Gender (male = 1)			7,943
Male	38.5		
Female	61.5		
Rural/urban residence (urban = 1)			7,943
Urban	55.9		
Rural	44.1		
R's reported years of schooling (mean)	2.0	3.6	6,855
R's household per capita income	14,454.8	14,540.1	5,772
**R's parental characteristics**			
Whether R often went to bed hungry in childhood			6,288
Yes	75.3		
No	24.7		
R's father's years of schooling (mean)	0.7	2.1	6,301

Some sub-categories may not add up to 100% due to rounding. R, respondent.

Source: Chinese Longitudinal Healthy Longevity Survey (CLHLS) 2018 data.

### 3.3. Methods

#### 3.3.1. Latent class analysis

Latent class analysis (LCA) was used to conduct the analyses in Stata 16.0 software to predict membership in latent or unobserved groups that share similar health lifestyle patterns among the Chinese oldest-old. LCA differs from factor analysis in that it uses dichotomous, not continuous, indicators and assumes that there are underlying discrete groups or classes of respondents. Membership in sub-groups is based on the similarities in individual responses to questions that are related to a set of observed behaviors. Latent classes of health lifestyles were created from the health lifestyle measures described in the previous section. Each case was assigned a probability of membership in each class. An exploratory approach was applied since the exact number of health behavior typologies is unknown. It started with the most parsimonious 1-class model and fitted successive models with increasing numbers of classes. Each latent class solution was replicated 20 times beginning at random starting values. This method included a close examination of item loadings and model fit indices for estimating latent classes (71).

The final number of classes was determined by the conceptual meaning, and commonly used fit measures such as the Akaike information criterion (AIC), the Bayesian Information Criterion (BIC), and the value of entropy. The AIC and BIC values for different class categories are presented in Table 2. The Stata software showed that convergence was not achieved when constructing four classes. Therefore, the study only presented the AIC, BIC, and entropy values for the first three classes. Since smaller values of AIC and BIC are better, the three-class model was considered the best fit. The entropy for the three-class model (0.703) is beyond the criteria for a good class separation cutoff point of 0.60 (72). The three-class solution also provided the most conceptually coherent description of health lifestyles. It was therefore chosen as the most appropriate solution. **Table 4** shows item response probabilities and shares for the sample for each class.

**Table 2:** Summary of latent class model identification and statistics (*N* = 6,553).

**No. of classes**	**AIC**	**BIC**	**Entropy**	**Likelihood ratio chi-squared**
1	71,193.2	71,261.1	-	5,086.2
2	69,455.3	69,597.9	0.613	3,326.3
**3**	**68,432.3**	**68,649.5**	**0.703**	**2,281.3**

AIC, Akaike's information criterion; BIC, Bayesian information criterion.

Bolded row represents the identified model.

#### 3.3.2. Other analyses

**Figure 1:**
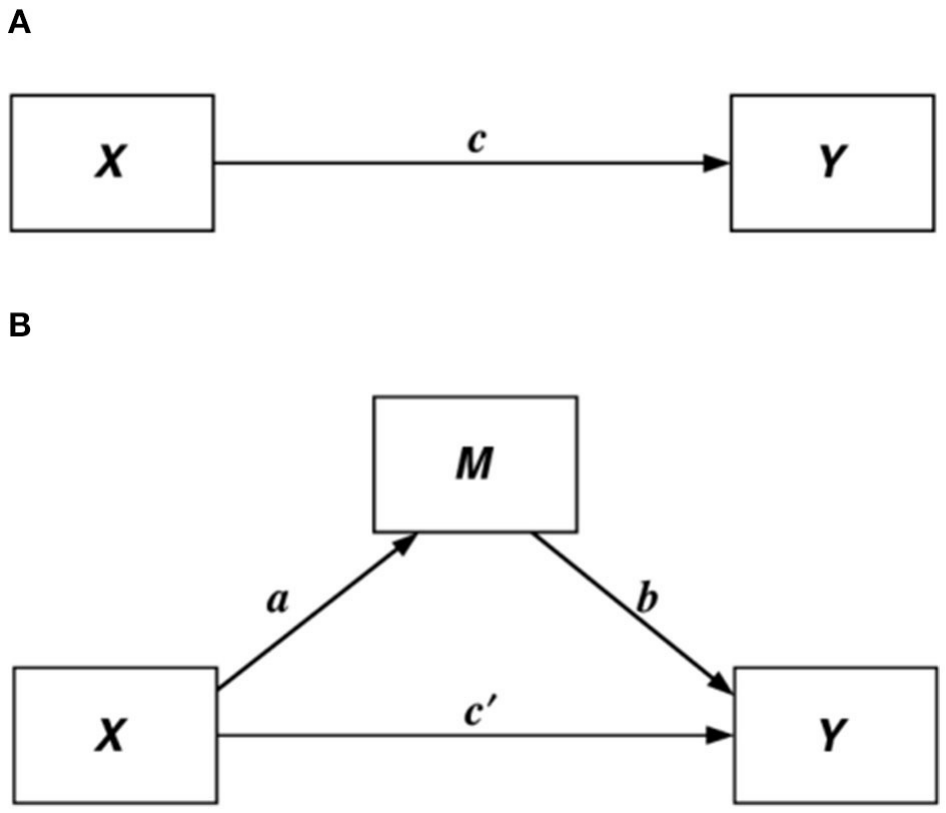
Illustration of a direct effect **(A)** and a mediation design **(B)**.

Descriptive analysis was used to report means and percentage distributions of all variables. To investigate whether health lifestyles served as a mediator in the association between living arrangements and Chinese oldest-old health, the study constructed several regression models: (1) ordinary least square (OLS) regression models detecting the association between living arrangements (X) and oldest-old health status (Y); (2) multinomial logit models showing how living arrangements (X) related to health lifestyle class membership, that is, oldest-old health lifestyles (M); and (3) adding health lifestyle covariates (M) into OLS regression models constructed in step (1) to evaluate whether health lifestyles served as a mediator. We followed Barton and Kenny's (73) rationale that has been widely used in social sciences to detect the existence of mediating effects. Specifically, if c, a, and b in Figure 1 were all significant, then a mediating effect existed. If adding M covariates made c' non-significant, then M had a perfect mediating effect (73, 74).

When using health lifestyles to predict the oldest-old health outcomes, multinomial logistic regression models were constructed. The multinomial logit regression equation is as follows:


$$\begin{array}{l} Logit\;k=logit\frac{{\text{Π}}_{k}}{{\text{Π}}_{n}}=\beta_{k}^{{}^{\prime }}X,K=1,2,\ldots \ldots ,n-1 \end{array}$$

where


$$\begin{array}{l} \begin{array}{l} \prod_{k}=\frac{\exp(\beta_{k}^{{}^{\prime }}X)}{1+\sum_{k=0}^{n-1}\exp(\beta_{k}^{{}^{\prime }}X)} \end{array} \end{array}$$

$$\begin{array}{l} \begin{array}{l} \prod_{n}=\frac{1}{1+\sum_{k=0}^{n-1}\exp(\beta_{k}^{{}^{\prime }}X)} \end{array} \end{array}$$

In multinomial logit models, the dependent variable had three categories or classes. Class 3 was treated as the base category for comparison. **Table 5** shows the multinomial logistic regression results when comparing class 3 with the other two classes, respectively, controlling for the respondent's demographic and socioeconomic characteristics.

## 4. Results

### 4.1. Descriptive statistics

Table 1 shows descriptive results for all variables. Of the 7,943 respondents aged 85 to 105 years, 61.5% of them were females. The percentage share of urban respondents was higher than that of rural ones (55.9% and 44.1%, respectively). The mean age of the sample was 94.7 with a standard deviation of 5.9. The SES of the studied sample appeared to be low. On average, the years of schooling among the studied sample was 2.0 with a standard deviation of 3.6. The mean household per capita income for the year before the survey was 14,454.8 RMB (which is equivalent to 2,065.0 USD with 1 USD = 7 RMB), with a standard deviation of 14,540.1 RMB. About 75.3% of the studied sample reported being hungry when going to bed in their childhood. The paternal socioeconomic status was even lower with an average year of paternal schooling of 0.7 with a standard deviation of 2.1.

As to health outcome variables, the SRH was fairly good with a mean score of 3.4 (between fair and good). The mean cognitive function score was 26.8, indicating good cognitive function considering the maximum cognitive function score was 30. The positive feeling score ranged from 5 to 25 and the negative feeling score ranged from 17 to 71. The mean positive and negative feeling scores were 12.2 and 29.0, respectively, suggesting that the sampled Chinese oldest-old had fairly positive subjective wellbeing.

For health lifestyle measures, the results showed that 21.1% and 41.6% of the respondents reported that they ate fresh fruits and vegetables almost every day and 12.3% of the oldest-old drank tea almost every day. About 10.2% of the studied sample were smokers and still smoked when the survey was conducted. In total, 9.7% of the respondents were drinkers and were still drinking in the survey year. About half of the respondents reported good quality sleep, and about 53.5% of them had 8 or more h of sleep each day. The results showed that 15.9% of the oldest-old reported that they did physical exercise before the age of 60 years and were still exercising when surveyed. About 27.1% and 40.4% of the respondents answered that almost every day they participated in at least one physical and sedentary type of leisure activity, respectively.

Table 3 shows the living arrangement patterns of the sampled oldest-old. The oldest-old who co-resided only with their son(s) or grandson(s) were the majority of the sample (47.2%). Such a percentage was higher among rural than among urban respondents (53.1% vs. 42.5%). Those who lived alone were the second largest group, which accounted for 16.3% of the overall sample (18.4% for urban vs. 14.6% for rural). It was followed by the group who lived only with daughter(s) or granddaughter(s) and the group who lived only with a spouse, which were 10.8% and 10.1% of the overall sample, respectively. The percentage of those who lived only with daughter(s) or granddaughter(s) was higher among urban oldest-old (12.5%) than among rural ones (8.7%). There was a slightly higher percentage of respondents who co-resided with a spouse in urban than in rural settings (10.6% vs. 9.5%). The other types of living arrangement patterns were not popular. About 7.7% of urban oldest-old reported living in nursing homes and the corresponding percentage for their rural counterparts was only 1.7%.

**Table 3:** Residential arrangements of oldest-old in China (%).

**Variable**	**Urban (*N* = 4,373)**	**Rural (*N* = 3,444)**	**Total (*N* = 7,817)**
**Living with a spouse only**	10.6	9.5	10.1
**Living with adult children or grandchildren, no spouse**			
Son or grandson only	42.5	53.1	47.2
Daughter or granddaughter only	12.5	8.7	10.8
Both son/grandson and daughter/granddaughter	3.2	3.1	3.2
**Living with spouse and adult children/grandchildren**			
Son/grandson only	4.1	4.1	4.1
Daughter/granddaughter only	1.1	0.5	0.8
Both son/grandson and daughter/granddaughter	0.4	0.1	0.3
**Living with no spouse, no adult children**			
Nursing home	7.7	1.7	5.0
With others	3.3	0.8	2.2
**Living Alone**	14.6	18.4	16.3

Some sub-categories may not add up to 100% due to rounding.

Chinese Longitudinal Healthy Longevity Survey (CLHLS) 2018 data.

### 4.2. The health lifestyles among Chinese oldest-old

Since the 3-class model was chosen as the best-fitted latent class model, the study estimated item probabilities for three identified latent classes. Table 4 presents the three predominant healthy lifestyles (latent classes) among the Chinese oldest-old and their share of the sample. Class 1 can be described as having a less healthy diet, not smoking, not drinking, poor sleep, and low engagement in physical exercise and leisure activities, which contained 45.0% of the sampled Chinese oldest-old in this group and may be labeled as having “negative behaviors”. This group showed that there were very low probabilities of respondents eating fruits or drinking tea every day. They were more likely to be non-smokers or non-drinkers both currently and during the past, and they had poor sleep and reported low engagement in leisure activities and physical exercise. This class was the majority among the three classes.

**Table 4:** Item response probabilities for health lifestyle indicators used in latent class analysis: Chinese oldest-old aged 85–105.

	**Class 1**	**Class 2**	**Class 3**
**Variables**	**(Less healthy diet, not smoking, not drinking, poor sleep, low physical exercise and leisure activities; 45.0%)**	**(Less healthy diet, not smoking, not drinking, good sleep, low physical exercise and leisure activities; 25.9%)**	**(Consistent engagement in healthy behaviors; 29.1%)**
**Health lifestyle indicators**			
**1. Eating fresh fruit almost everyday**			
Yes	0.092	0.189	0.425
No	0.908	0.811	0.575
**2. Eating fresh vegetables almost everyday**			
Yes	0.462	0.563	0.839
No	0.538	0.437	0.161
**3. Drinking tea almost everyday**			
Yes	0.062	0.093	0.278
No	0.938	0.907	0.722
**4. Smoking**			
Yes	0.069	0.100	0.188
No	0.931	0.900	0.812
**5. Drinking**			
Yes	0.065	0.094	0.181
No	0.0.935	0.906	0.819
**6. Good quality of sleep**			
Good	0.227	0.937	0.636
Poor	0.773	0.063	0.364
**7. Normally sleeps at least 8 h**			
Yes	0.234	0.999	0.560
No	0.766	0.001	0.440
**8. Exercising during the past and at present**			
Yes	0.079	0.100	0.406
No	0.921	0.900	0.594
**9. Participating in physical leisure activities almost daily**			
Yes	0.270	0.175	0.475
No	0.730	0.825	0.525
**10. Participating in sedentary leisure activities almost daily**			
Yes	0.281	0.286	0.856
No	0.719	0.714	0.144

All variables are coded 1 = yes, 0 = no.

Sources: Chinese Longitudinal Healthy Longevity Survey (CLHLS) 2018 data.

About 25.9% of the respondents fell into Class 2, which can be described as having a less healthy diet, not smoking, not drinking, good sleep, and low engagement in physical exercise and leisure activities. This group may be labeled as having “adequate sleep”. For this group, the probabilities of respondents drinking tea and eating fresh fruits/vegetables were slightly higher as compared to the first group. They were not smokers or drinkers previously or at the survey time and had low probabilities of exercising and participating in leisure activities. A dominant feature of this class was that this class had the best sleep among all three groups.

Class 3 can be classified as consistent engagement of healthy behaviors, which accounted for 29.1% of the studied sample and may be labeled as having “healthy behaviors”. This class showed the highest probabilities of having healthy dietary patterns (eating fresh vegetables and fruits almost every day; drinking tea almost every day), not being smokers or drinkers, having enough sleep (≥8 h per day), and reporting good quality sleep, participating in active and sedentary leisure activities and doing physical exercises. Based on the above results, class 3 was labeled as consistently positive by showing overall healthier lifestyles relative to nearly all measures and domains. The other two classes are composed of particular domains of unhealthy behaviors.

### 4.3. Living arrangements and Chinese oldest-old health lifestyles

The study then constructed multinomial logistic regression models to investigate the associations between oldest-old living arrangement patterns and their health lifestyles, i.e., how living arrangements predicted health lifestyles. Class 3, the consistently positive group, was treated as the baseline group and the other two classes were compared with class 3. Results presented in Table 5 show that respondents who lived only with a spouse and those who lived with a spouse and children/grandchildren were more likely to be in the other two classes than in class 3. Those who lived alone were more likely to be in class 1 (less healthy diet, not smoking, not drinking, poor sleep, and low engagement in physical exercise and leisure activities) than in class 3. These findings indicated that the Chinese oldest-old who lived only with children/grandchildren were more likely to have healthier lifestyles than those who co-resided only with a spouse, those living with a spouse and children/grandchildren together, and those who lived alone.

**Table 5:** Multinomial logistic regression on R's health lifestyle latent classes: Chinese oldest-old aged 85–105.

**Variables**	**Class 1 vs. class 3**		**Class 2 vs. class 3**	
	**RRR**	**S.E**.	**RRR**	**S.E**.
**R's living arrangement patterns**				
**(Ref**.= **living with children or grandchildren only, no spouse)**				
Living with a spouse only	1.58^***^	0.14	1.56^**^	0.14
Living with spouse and children/grandchildren	1.41^***^	0.18	1.49^**^	0.17
Living alone	1.89^**^	0.21	1.09	0.25
Living in nursing homes	1.20	0.51	0.35	0.70
Living with others	1.38	0.25	1.50	0.25
**Control variables**				
Age	0.95^***^	0.01	0.90^***^	0.01
Sex (Ref. = female)	0.66^***^	0.09	1.34^**^	0.10
Residence (Ref. = rural)	0.65^*^	0.08	1.58^***^	0.09
R's years of schooling	0.99	0.02	1.06^***^	0.01
R's natural logged per capita family income	0.92^**^	0.03	1.14^***^	0.03
If R often when to bed hungry in childhood	0.89	0.10	0.79^*^	0.10
R's father's years of schooling	0.99	0.02	1.0	0.02
Constant	5.30^***^	0.73	7.20^***^	0.81
N	4,223		4,223	
LR Chi- squared	654.65		654.65	
Log likelihood	−4,292.02		−4,292.02	

R refers to the respondent; ^*^represents p < 0.05; ^**^p < 0.01; ^***^p < 0.001. RRR, relative risk ratio.

Sources: Chinese Longitudinal Healthy Longevity Survey (CLHLS) 2018 data.

As to control variables, the results indicated that with age increasing, the Chinese oldest-old were less likely to be in the other two classes than in class 3, suggesting they tended to have healthier lifestyles while aging. As compared to women, men were less likely to be in class 1 but more likely to be in class 2 (less healthy diet, not smoking, not drinking, good sleep, and lowest engagement in physical exercise and leisure activities) than in class 3. Urban seniors were less likely to be in class 1 but more likely to be in class 2 than in class 3 as compared to their rural counterparts. Higher education and family income pushed the oldest-old to be more likely in class 2. Respondents who frequently went to bed hungry in childhood were less likely to be in class 2 than in class 3. These results demonstrated that demographic features as well as individual and parental SES significantly linked to the Chinese oldest-old health lifestyle class membership, that is, as compared to class 3, socioeconomically disadvantaged groups were more likely to be in class 1 but the oldest-old with higher SES tended to have a greater likelihood of being in class 2. This meant that higher SES does not always lead to healthier lifestyles. The sampled Chinese oldest-old with higher SES indeed tended to fall into class 2 which has an unhealthy diet, sedentary lifestyle, and good sleep quality. These findings will be discussed in the discussion section.

### 4.4. The influence of living arrangements and health lifestyles on Chinese oldest-old health

This section of the analysis started to follow Baron and Kenny's (73) strategy by performing regression analyses to detect if health lifestyles served as a mediator in the association between living arrangements and Chinese oldest-old health. The study first performed OLS regression in models 1, 3, 5, and 7 by using living arrangements to predict the respondent's health status which was measured by SRH, cognitive function, and positive and negative feelings, respectively. Models 2, 4, 6, and 8 further added health lifestyle covariates in OLS regression models to discover if health lifestyles served as a mediator linking living arrangements and the oldest-old health (see Tables 6A, B).

**Table 6A:** OLS regression of self-rated health and cognitive function on living arrangements, health lifestyle latent classes and other control variables: Chinese oldest-old aged 85–105.

	**Self-rated health**				**Cognitive function**			

**Variables**	**Model 1**		**Model 2**		**Model 3**		**Model 4**	
	**b**	***p*** **value**	**b**	***p*** **value**	**b**	***p*** **value**	**b**	***p*** **value**
**R's living arrangement patterns (Ref**.= **living with children or grandchildren, no spouse)**								
Living with a spouse only	−0.22	0.000	−0.20	0.000	0.46	0.138	0.41	0.189
Living with spouse and children/grandchildren	−0.00	0.961	−0.00	0.986	0.53	0.193	0.47	0.252
Living alone	−0.01	0.868	0.04	0.574	0.22	0.711	0.22	0.700
Living in nursing homes	0.11	0.585	0.19	0.339	−0.27	0.875	−0.34	0.841
Living with others	0.06	0.540	0.06	0.494	−0.40	0.540	−0.43	0.504
**Health lifestyle latent class (Ref**.= **Class 3: Consistent engagement in healthy behaviors)**								
Class 1 (less healthy diet, not smoking, not drinking, poor sleep, low physical exercise and leisure activities)	-	-	−0.29	0.000	-	-	0.30	0.354
Class 2 (less healthy diet, not smoking, not drinking, good sleep, lowest physical exercise and leisure activities)	-	-	0.20	0.000	-	-	0.81	0.005
**Control Variables**								
Age	−0.00	0.522	0.00	0.928	−0.13	0.000	−0.12	0.000
Sex (Ref. = female)	0.03	0.366	−0.03	0.463	0.71	0.007	0.70	0.008
Residence (Ref. = rural)	0.01	0.823	−0.01	0.677	0.09	0.712	0.01	0.988
R's years of schooling	−0.01	0.723	−0.01	0.117	0.09	0.009	0.07	0.020
R's natural logged per capita family income	0.07	0.000	0.06	0.000	0.22	0.020	0.19	0.037
If R often when to bed hungry in childhood	−0.07	0.047	−0.07	0.027	−0.34	0.167	−0.34	0.171
R's father's years of schooling	−0.02	0.044	−0.02	0.000	0.02	0.703	0.02	0.655
Constant	3.04	0.000	3.05	0.000	36.24	0.000	35.34	0.000
N	3,680				1,165			

^*^ < 0.05, ^**^ < 0.01, ^***^ < 0.001. R, respondent. b, regression coefficient; S.E., standard error.

Sources: Chinese Longitudinal Healthy Longevity Survey (CLHLS) 2018 data.

**Table 6B:** OLS regression of subjective wellbeing on living arrangements, health lifestyle latent classes and other control variables: Chinese oldest-old aged 85–105.

	**Positive wellbeing**				**Negative wellbeing**			

**Variables**	**Model 5**		**Model 6**		**Model 7**		**Model 8**	
	**b**	***p*** **value**	**b**	***p*** **value**	**b**	***p*** **value**	**b**	***p*** **value**
**R's living arrangement patterns (Ref**.= **Living with children or grandchildren, no spouse)**								
Living with a spouse only	0.45	0.002	0.57	0.003	0.10	0.818	−0.02	0.961
Living with spouse and children/grandchildren	0.40	0.020	−0.08	0.744	−0.38	0.518	−0.42	0.466
Living alone	0.09	0.727	0.08	0.808	1.72	0.019	1.30	0.072
Living in nursing homes	−1.06	0.129	0.08	0.925	2.40	0.189	1.79	0.318
Living with others	0.41	0.105	−0.22	0.555	1.38	0.101	1.20	0.146
**Health lifestyle latent class (Ref**.= **Class 3: Consistent engagement in healthy behaviors)**								
Class 1 (less healthy diet, not smoking, not drinking, poor sleep, low physical exercise and leisure activities)	-	-	−0.29	0.000	-	-	2.52	0.000
Class 2 (less healthy diet, not smoking, not drinking, good sleep, lowest physical exercise and leisure activities)	-	-	0.20	0.000	-	-	−0.88	0.013
**Control variables**								
Age	−0.00	0.522	0.00	0.928	−0.05	0.037	−0.06	0.020
Sex (Ref. = female)	0.03	0.366	−0.03	0.463	−1.16	0.000	−0.73	0.024
Residence (Ref. = rural)	0.01	0.823	−0.01	0.677	0.51	0.085	0.64	0.027
R's years of schooling	−0.01	0.723	−0.01	0.117	−0.07	0.127	−0.03	0.485
R's natural logged per capita family income	0.07	0.000	0.06	0.000	−0.52	0.000	−0.42	0.000
If R often when to bed hungry in childhood	−0.07	0.047	−0.07	0.027	0.71	0.034	0.70	0.032
R's father's years of schooling	−0.02	0.044	−0.02	0.000	0.03	0.662	0.03	0.605
Constant	3.04	0.000	3.05	0.000	38.24	0.000	37.03	0.000
N	3,680				2,782			

b, regression coefficient; S.E., standard error.

Sources: Chinese Longitudinal Healthy Longevity Survey (CLHLS) 2018 data.

Models 1 and 2 in Table 6A showed that the average SRH score for the oldest-old who lived only with a spouse was 0.22 lower than those who lived only with children/grandchildren. The significant regression coefficient for health lifestyle covariates was significant as well, suggesting that as compared to class 3, the oldest-old in class 1 tended to report worse SRH but respondents in class 2 tended to report better SRH scores. When adding health lifestyle classes in model 2, the regression coefficient became smaller, indicating health lifestyles served as a mediator which partially explained the association between living arrangements and SRH.

Models 3 and 4 presented regression results when predicting the cognitive function status of the Chinese oldest-old. Although cognitive function scores for the oldest-old in class 2 were 0.81 points higher than that of the reference group, class 3, living arrangements did not show significant effects on respondents' cognitive function. Thus, no mediating effect existed.

The results in models 5 and 6 in Table 6B showed that the oldest-old who only lived with a spouse and those who lived with a spouse and children together tended to report higher positive wellbeing scores than those who lived only with children/grandchildren. The coefficients for health lifestyles covariates were significant. When adding health lifestyle classes in model 6, the significant health disparities between the reference group and the group who lived with a spouse and children/grandchildren disappeared. It indicated that the mediating effect of health lifestyles existed, which perfectly explained the health disparities caused by living arrangement differences, but the oldest-old who lived only with a spouse still showed higher positive wellbeing scores than the reference group, even after health lifestyles and other factors were controlled.

Models 7 and 8 further showed regression results when predicting the oldest-old negative wellbeing scores. Those who lived alone showed a significantly higher negative wellbeing score than the reference group in model 7. Adding health lifestyle memberships in model 8 made such a significant effect disappear. It indicated a medicating effect of health lifestyles, i.e., health lifestyles perfectly explained the significant differences in negative wellbeing scores between the reference group and the group who lived alone. The health lifestyle covariates showed significant effects. Class 1 tended to have a negative wellbeing score of 2.5 points higher than the reference group; whereas class 2 tended to report a score of 0.88 lower than class 3.

Control variables showed significant correlations with the respondent's health. An increasing age is related to a worse cognitive function and a lower negative wellbeing score. Male participants tended to have better cognitive function and better subjective wellbeing. The urban oldest-old seemed to have a higher negative wellbeing score than their rural counterparts. Higher education was linked to better cognitive function and a higher positive wellbeing score. High income showed significant positive effects on SRH, better cognitive function, and subjective wellbeing. Going to bed hungry in childhood had significantly negative effects on the oldest-old SRH and subjective wellbeing. Such findings supported the cumulative disadvantage theories that childhood disadvantage explained part of the health disparities in older ages. In sum, the findings of this research proved that some of the health differentials linked to the oldest-old living arrangement patterns can be explained by their health lifestyles in various household contexts. In other words, health lifestyles served as a factor mediating the association between living arrangements and Chinese oldest-old health status, after controlling for demographic and socioeconomic covariates.

## 5. Conclusion and discussion

Through analyzing samples aged 85 to 105 years from data of the CLHLS 2018 wave, the research tried to fill the voids of prior literature by elucidating the mediating effect of health lifestyles. Three latent classes representing three predominant health lifestyles among Chinese oldest-old emerged, which were labeled as groups having “negative behavior”, “adequate sleep”, and “constant positive behaviors”, respectively. Individuals in class 1 accounted for nearly half of the studied sample, which represented the most popular health lifestyles of the Chinese oldest-old. Only about one-third of the respondents demonstrated healthy lifestyles.

Nearly half of the oldest-old were found to live only with son(s)/grandson(s), which was the most popular living arrangement pattern, followed by living alone, living only with daughter(s)/granddaughter(s), and living only with a spouse. The rest types of living arrangements were uncommon. The results were consistent with previous findings that co-residing with one or more adult children is still a fundamental household structure in China (15). Living arrangement patterns were also found to be significantly correlated with the oldest-old health lifestyles. As compared to those who lived only with adult children/grandchildren, those who lived only with a spouse, and those who lived with a spouse and child/grandchildren together were more likely to be in class 1 (having poor sleep, unhealthy diet, and a sedentary lifestyle) and class 2 (having good sleep, unhealthy diet, and a sedentary lifestyle) than in class 3. Those who lived alone were also more likely to be in class 1 than in class 3. These results suggested that besides having an unhealthy diet and a sedentary lifestyle, the oldest-old who lived alone were especially more likely to experience poor sleep. Such results fell in line with findings of prior literature that Asian older adults who lived with family members had greater odds of reporting good quality of sleep and a longer duration of sleep (20, 75). The oldest-old living only with son(s)/grandson(s) were more likely to be in class 3 and have healthier lifestyles than those in other types of household contexts. Living with a spouse did not seem to aid older adults in forming healthier lifestyles.

When predicting health outcomes, in addition to cognitive function, living arrangements were found to have significant correlations with the respondent's health. Specifically, as compared to the oldest-old who co-resided only with their children or grandchildren, those living only with a spouse reported worse SRH. Individuals who lived only with a spouse and those who lived with a spouse and children/grandchildren together reported higher positive wellbeing scores than those who lived only with son(s)/grandson(s). Living alone significantly increased the respondent's negative wellbeing score. It seemed that co-residing with a spouse is a critical factor that promoted the oldest-old positive wellbeing, whereas the absence of other family members significantly deteriorated elders' subjective wellbeing.

Health lifestyles were found to serve as a mediator that partially explained the respondent's SRH disparities. That is, the oldest-old who lived only with a spouse had a worse SRH score because they had unhealthy lifestyles than those who co-resided only with son(s)/grandson(s). Health lifestyles thus partially explained SRH differentials. Health lifestyles did not show a mediating effect when predicting cognitive function. Regarding positive wellbeing, when adding health lifestyles in the model, although the oldest-old living only with a spouse still showed significantly higher positive feeling scores than those who lived only with son(s)/grandson(s), the health advantage of those who lived with a spouse and children/grandchildren disappeared. The findings implied that no matter whether the respondent lived with a spouse or not, co-residing with adult children/grandchildren helps older adults to shape healthy lifestyles, which perfectly explains the differentials in positive feelings caused by living arrangements.

The mediating effect of healthy lifestyles may be explained as follows: First, adult children/grandchildren have more updated health-related knowledge than the oldest-old and their spouse who is most likely to be an oldest-old individual as well. Adult children/grandchildren who lived with the oldest-old are also more capable of facilitating their elder parents/grandparents to practice healthy behaviors than the respondent's spouse. Second, it is possible that those living alone are also likely to have sedentary, isolated, and unhealthy lifestyles, which may have caused their worse health condition and stronger negative feelings. Thus, health lifestyles serve as a mediator in this study.

The literature review section reviewed five theories to elucidate how living arrangements may possibly explain the health disparities among older adults with various living arrangement patterns. The mediating effects discovered in this research inform the reader that health lifestyles may serve as a theoretical explanation of the above association. It can be named *healthy lifestyles as a mediator explanation* of the health disparities among the oldest-old in different household contexts. This finding not only enriched the existing theories on older adults' living arrangements and health but also had important practical implications. Caregivers, clinicians, and professionals may consider assisting older adults to form healthier lifestyles to improve their health and longevity. The research results echoed the arguments of researchers that multiple health behavior change interventions outperformed single-behavior interventions in health promotion (76, 77). Results based on analyzing the China data also provided valuable implications to address disease prevention and health promotion-related issues among older adults in other countries.

It was found that the oldest-old living only with son(s)/grandson(s) generally had a greater chance to be in class 3 (“having constant positive behaviors”) than those living in other types of household structures, suggesting that the oldest-old living with son(s)/grandson(s) tended to have healthier lifestyles. The *Filial piety theory* contends that living with married sons benefits older adults' health, especially psychological wellbeing (27, 28). The findings of this research, however, challenged this theory by showing being in class 3 did not lead to the best health outcomes. Instead, the oldest-old in class 2 reported better SRH, cognitive function, and subjective wellbeing than those in class 3. Higher SES among individuals in class 2 likely contributed to their better health outcomes. Regression results showed that the oldest-old with higher income and education were more likely to be in class 2 (“adequate sleep group”) than in class 3. Their high SES somehow offsets the negative effects of unhealthy lifestyles on health. This group therefore demonstrated the best health outcomes among all three classes. Such findings can be explained by China's current transitional stage in which consuming high-fat and energy-condensed food and having a more sedentary lifestyle are considered a privilege of people with higher SES (78). Thus, the oldest-old in class 2 had less healthy lifestyles but better health outcomes. Positive links between higher SES, healthier lifestyles, and better health outcomes are expected to occur among individuals in China after the country completes its social and economic transitions in years to come.

The study had several limitations. First, the research was not able to exhaust all possible health lifestyle measures due to limited CLHLS survey questions relating to health lifestyles. Some important health lifestyle indicators, such as vaccination injections and doctoral visits, have not been included in this research. Second, measures of one's health status were also relatively crude. Future research may consider applying additional health outcomes as well as health lifestyle measures to improve current analysis. In addition, the quality of living arrangements and duration of stay in certain household contexts were not controlled in the analysis due to data constraints. Since the quality of care provided in the household may differ among the oldest-old with the same living arrangement type and the duration of stay in certain living arrangement patterns may link to the oldest-old health, future research should consider these dimensions. Finally, although the study showed a mediating effect of health lifestyles, there may be a causality issue between health lifestyles and health outcomes. It could be the case that healthier individuals are more likely to have healthier lifestyles, which in turn benefits one's health. Future research should further address the causality issue to yield a more comprehensive understanding of living arrangements, health lifestyles, and the oldest-old health outcomes.

## Data availability statement

Publicly available datasets were analyzed in this study. This data can be found here: https://opendata.pku.edu.cn/file.xhtml?fileId=10357.

## Author contributions

LiZ and JW were major contributors. LiZ designed the research, conducted literature review, analyzed data, and wrote the original draft. JW conducted literature review and formal analysis and revised the manuscript. LaZ and SW conducted literature review, analyzed and interpreted data, and revised the manuscript. All authors read and approved the final manuscript.
